# Letter to the editor regarding the article ‘EFSA’s toxicological assessment of aspartame: was it even-handedly trying to identify possible unreliable positives and unreliable negatives?’

**DOI:** 10.1186/s13690-020-0395-4

**Published:** 2020-04-02

**Authors:** George E. N. Kass, Federica Lodi

**Affiliations:** 1 Scientific Committee and Emerging Risks Unit, European Food Safety Authority, 43126 Parma, Italy; 2 Food Ingredients and Packaging Unit, European Food Safety Authority, 43126 Parma, Italy

**Keywords:** Aspartame, Sweetener, Risk assessment

## Abstract

This letter is in response to a recent paper by Millstone and Dawson (2019) in which the authors criticise the re-evaluation of the high intensity sweetener aspartame in 2013 by the former EFSA’s Panel on Food Additives and Nutrient Sources added to Food, on the grounds that EFSA did not follow its own procedures for its risk assessment. Moreover, the authors claim that the appraisal of the available studies was asymmetrically more alert to putative false positives than to possible false negatives. In this letter it is shown that the methodology for collection and selection of the scientific information used as a basis for the aspartame risk assessment, and the inclusion/exclusion criteria applied were defined a priori and documented in the published opinion. Furthermore, the Panel applied a Weight-of-Evidence approach combined with an analysis of the biological relevance of the appraised and validated evidence for its analysis, integration and interpretation, followed by an uncertainty analysis. Finally, an analysis of the distribution of negative versus positive outcome of the studies in the context of reliability showed that the claim of bias in the scientific risk assessment of aspartame is not substantiated.

## Main text

We thank Millstone and Dawson for their article on EFSA’s toxicological assessment of aspartame [1]. EFSA fully agrees that scientific assessments should be evidence-based, and that a structured and documented approach describing the methodologies to collect, select, evaluate and integrate scientific evidence, defined before-hand is essential. Likewise, essential are transparent and open communication of the processes and results to decision makers, the wider scientific community and stakeholders. Such considerations have always been an integral feature of the operations of EFSA [2]. Several guidance documents and EFSA’s PROMETHEUS project (PROmoting METHods for Evidence Use in Scientific assessments) have been developed to support the robustness, transparency and openness of its scientific assessments [3, 4].

For the re-evaluation of aspartame in 2013, the former EFSA Panel on Food Additive and Nutrient Sources added to Food (ANS), based its assessment on data and evidence coming from public calls for data and from extensive literature searches [5]. In addition, previous scientific evaluations by national and international agencies and independent expert advisory committees were considered. The methodology (defined a priori) for the collection and consideration of the scientific information/data for the risk assessment is detailed in Appendix A of [6]. Most studies were conducted before OECD test guidelines and GLP (Good Laboratory Practice) measures were implemented in regulatory toxicology. However, these were considered on a case-by-case basis if their design and the reporting of the data were considered appropriate [6]. EFSA’s assessment of aspartame was based on the available data, relying mainly on primary studies, and was not limited to a simple endorsement of previous assessments or on third party anecdotal evidence from e.g. memos, letters or US congressional hearings. Furthermore, the ANS Panel applied a clearly documented Weight-of-Evidence approach combined with an analysis of the biological relevance of the appraised and validated evidence for its analysis, integration and interpretation (Section 13 of [6]). This included an uncertainty analysis. Overall, and in contrast with the claims in [1], these elements demonstrate that the approach followed by the ANS Panel was a rigorous one, ensuring an independent and in-depth analysis of all existing data [6].

In their recent publication, Millstone and Dawson (2019) claimed that the ‘*the panel’s appraisal of the available studies was asymmetrically more alert to putative false positives than to possible false negatives*’ and that this ‘*bias would have favoured commercial interests to the detriment of consumer protection’.* They state that EFSA judged 62 out of 81 of the ‘negative’ studies, i.e. those not showing any adverse effect, to be reliable. In contrast, every putative ‘positive’ study, i.e. showing an adverse effect, was stated to have been evaluated by EFSA as unreliable (Table 1). In other words, the claim is that the ANS Panel discounted 100% of the evidence of harm.

**Table 1:** Studies not indicating adverse effects (‘negative’ studies)^a^*versus* studies indicating possible adverse effects (‘positive’ studies)^a^

	Number of studies identified	Treated as reliable	Treated as unreliable
**Studies not indicating adverse effects (‘negative’ studies)**^**a**^			
Millstone and Dawson^b^	81	62 (77%)	19 (23%)
EFSA^c^	78	51 (65%)	27 (35%)
**Studies indicating possible adverse effects (‘positive’ studies)**^**a**^			
Millstone and Dawson^b^	73	0 (0%)	73 (100%)
EFSA^c^	37	21 (57%)	16 (43%)

^a^ Only primary sources of information relevant to the risk assessment process were included by EFSA in the analysis

^b^ Interpretation by Millstone and Dawson of the way how the ANS Panel had judged studies as reliable or not reliable

^c^ Interpretation by EFSA of the way how the ANS Panel had judged studies as reliable or not reliable. The criteria are described in the text, and information on the individual studies is detailed in [6]

EFSA has performed an analysis of the distribution of ‘negative’ versus ‘positive’ studies on the toxicological properties of aspartame and their reliability. EFSA’s analysis took into consideration the limitations of the studies, the biological relevance of the observations reported and the reliability (overall or partial) and use of the evidence in the risk characterisation process. Information on the individual studies considered is detailed in [6]. The outcome of EFSA’s analysis of the scientific studies clearly shows that the ANS Panel had considered over one third of the ‘negative’ studies as unreliable and over half of the ‘positive’ studies as reliable (Table 1). Therefore, EFSA refutes the claim of bias in the assessment of ‘negative’ versus ‘positive’ studies.

## Data Availability

Not applicable.

## Abbreviations

ANS Panel: Panel on Food Additives and Nutrient Sources added to Food
EFSA: European Food Safety Authority
GLP: Good Laboratory Practice

## References

[1] Millstone EP, Dawson E (2019). EFSA’s toxicological assessment of aspartame: was it even-handedly trying to identify possible unreliable positives and unreliable negatives?. Arch Publ Health.

[2] EFSA Scientific Committee (2015). Guidance on the review, revision and development of EFSA’s cross-cutting guidance documents. EFSA J.

[3] EFSA Scientific Committee (2009). Guidance of the scientific committee on transparency in the scientific aspects of risk assessments carried out by EFSA. Part 2: general principles. EFSA J.

[4] EFSA (European Food Safety Authority) (2015). Editorial: increasing robustness, transparency and openness of scientific assessments. EFSA J.

[5] Call for scientific data on Aspartame (E951) (published: 1 June 2011). Available online: http://www.efsa.europa.eu/en/dataclosed/call/110601.htm; Call for scientific data on aspartame (E 951) related to 5-benzyl-3,6-dioxo-2-piperazine acetic acid (DKP) and other primary or secondary degradation products from aspartame (published: 26 July 2012). Available online: http://www.efsa.europa.eu/en/dataclosed/call/120726.htm.

[6] EFSA ANS Panel (EFSA Panel on Food Additives and Nutrient Sources added to Food) (2013). Scientific opinion on the re-evaluation of aspartame (E 951) as a food additive. EFSA J.

